# Efficacy of Jihwangeumja (Dihuang Yinzi) on cognitive function and activities of daily living in patients with Alzheimer disease: A protocol for a systematic review and meta-analysis

**DOI:** 10.1097/MD.0000000000025592

**Published:** 2021-05-14

**Authors:** Jae Yeong Lee, Ju Yeon Kim, Ji-Yoon Lee, Jin-Hyeong Jung, In Chul Jung

**Affiliations:** a College of Korean Medicine, Daejeon University, Daejeon, Republic of Korea; b Department of Oriental Neuropsychiatry, College of Korean Medicine, Daejeon University, Daejeon, Republic of Korea.

**Keywords:** Alzheimer disease, dementia, Jihwangeumja, protocol, systematic review

## Abstract

**Background:**

This systematic review protocol describes the methods proposed to evaluate the efficacy and safety of Jihwangeumja in patients with Alzheimer disease.

**Methods:**

The following databases, PubMed, EMBASE, CENTRAL, Cumulative Index to Nursing and Allied Health Literature, China National Knowledge Infrastructure, National Digital Science Library, Korean Information Service System, and Korean Medical Database will be searched for relevant publications without language or publication status restrictions. Search terms will be based on “Alzheimer” for participants and “Jihwangeumja” or “Dihuang Yinzi” for interventions. Two researchers will independently extract the study data from the included studies and only randomized controlled trials will be included. The risk of bias will also be assessed independently by 2 researchers using the Cochrane risk of bias tool. We will use RevMan software random-effects and fixed-effect models for the assessment of heterogeneity and data synthesis. Any changes in the plan for documenting significant protocol amendments will require the researchers to have a revision agreement and register the international prospective register of systematic review modification.

**Results:**

The treatment effect and safety will be measured by meta-analysis and the quality of the included studies will be reviewed.

**Conclusion:**

This systematic review will provide evidence regarding the efficacy and safety of Jihwangeumja.

**Ethics and dissemination:**

Ethical approval is not required because individual patient data will not be included in this paper. The study findings will be disseminated through conference presentations.

**OSF Registration:**

DOI: 10.17605/OSF.IO/HXA58.

## 1 Introduction

Dementia is a condition where the usual activity of daily living (ADL) level of the patient cannot be maintained because of an acquired loss of cognition in multiple cognitive domains.^[1]^ As dementia progresses, patients cannot conduct their daily lives independently, which results in various burdens such as high treatment and the care cost, and the loss of labor for patients and their families.^[2]^

In 2019, more than 50 million people were diagnosed with dementia worldwide and with the rapid increase in the aging population, the number is expected to reach 152 million by 2050.^[3]^ The current annual cost of dementia is estimated to be US $1 trillion and is expected to double by 2030.^[3]^ Alzheimer disease (AD), the most common subtype of dementia, accounts for 55% to 70% of all dementia.^[4]^ The rapid increase in the aging population has increased the incidence of AD, which has placed a tremendous economic burden on society and patients’ families.^[5]^

The typical pathological findings of AD include extracellular amyloid β plaques and intracellular neurofibrillary tangles in the brain.^[6]^ AD patients consistently exhibit a significant loss of cholinergic neurons and reduced choline acetyltransferase activity, which is considered to contribute significantly to cognitive degradation.^[7,8]^ Accordingly, acetylcholinesterase inhibitors are used as the primary treatment for AD to prevent the enzymatic degradation of acetylcholine in the synaptic cleft.^[9]^ Unfortunately, these currently available chemical drugs have not exhibited the expected preventive or therapeutic effects.^[10]^ Instead of treating the underlying cause of AD or slowing cognitive decline, these medications only temporarily treat the symptoms or improve memory and cognition.^[11]^ Therefore, a new strategy for treating AD is urgently needed.

AD has been actively studied in Korean medicine and in 2017, a systematic review provided evidence supporting the therapeutic effects of herbal medicines on AD.^[12]^ Nevertheless, this study was based on various prescriptions, which has made its clinical application extremely limited. In 2017 and 2019, systematic reviews revealed the treatment efficacy of Choto-san and Danggui-Shaoyao-San prescriptions in patients with AD.^[13,14]^ Similarly, assessing the effects of a single prescription on patients with AD has helped increase the knowledge of clinicians. Therefore, we decided to review studies of single prescription treatments for AD.

Jihwangeumja is a prescription used to treat diseases caused by “kidney yin yang” total deficiency such as cerebral arteriosclerosis and aging-related stroke.^[15]^ In Korean medicine, kidney yin yang total deficiency is caused by aging and is considered a significant pathological factor of dementia. No clinical studies or systematic reviews have examined the clinical efficacy of Jihwangeumja for AD. Therefore, we intend to conduct a systematic review to summarize the clinical evidence of the efficacy of Jihwangeumja on cognitive function and the ADL in patients with AD. Furthermore, we will assess the safety of Jihwangeumja in the treatment of AD.

## 2 Methods

### 2.1 Protocol registration

This protocol for a systematic review has been registered on the international prospective register of systematic review with the registration number CRD42020220219. The protocol was strictly developed under preferred reporting items for systematic reviews and meta-analyses protocols.^[16]^

### 2.2 Eligible criteria for study selection

#### 2.2.1 Types of studies

We will include only randomized controlled trials assessing the efficacy and safety of Jihwangeumja on cognitive function and ADL in AD. Studies using parallel and crossover designs will be included, whereas other studies such as case reports, case series, literature review, and uncontrolled trials will be excluded. There will be no language restrictions during the database search, but all search words will be in English, Chinese, or Korean.

#### 2.2.2 Types of participants

Studies including participants with major neurocognitive impairment and who were diagnosed with AD will be included. There will be no restrictions on age, sex, nation, race, inpatient or outpatient, or medical history.

#### 2.2.3 Types of interventions

##### 2.2.3.1 Experimental interventions

Jihwangeumja in various formulas such as decoctions, capsules, granules, and powders will be included. Modified prescriptions consisting of the elemental compositions of Jihwangeumja (Rehmanniae Radix Preparata, Morindae Radix, Cornus officinalis Siebold et Zuccarini, Cistanche deserticola Y. C. Ma, Dendrobii Caulis, Polygalae Radix, Schisandra chinensis Baillon, Poria Sclerotium, Liriope platyphylla Wang et Tang, Aconiti Lateralis Radix Preparata, Cinnamomi Cortex, Acorus gramineus Solander, Menthae Herba, Zingiberis Rhizoma Recens, Zizyphi Fructus) will be included.^[17]^ Co-interventions with Western medications, herbal medicines, acupuncture, moxibustion, and cognitive training will be excluded because these may affect the overall therapeutic effects.

##### 2.2.3.2 Control interventions

Studies using Western medicine treatments or placebos as comparators will be selected. Other comparisons, such as those with add-on therapies with herbal medicines or acupuncture, will be excluded.

#### 2.2.4 Types of outcome measures

##### 2.2.4.1 Primary outcomes

The primary outcome measures are as follows:

(1) Cognitive function using assessments such as scores of mini-mental state examination, Hasegawa Dementia Scale, AD assessment scale-cognitive subscale, and Montreal cognitive assessment.

##### 2.2.4.2 Secondary outcomes

The secondary outcome measures are as follows:

(1) Barthel ADL index
(2) Instrumental ADL
(3) Modified Barthel Index
(4) Number and severity of adverse events
(5) Results of blood tests evaluating the association between Jihwangeumja and adverse events.

### 2.3 Search methods for identifying studies

#### 2.3.1 Electronic searches

The following 8 electronic databases will be searched without language restrictions: PubMed, EMBASE, CENTRAL, Cumulative Index to Nursing and Allied Health Literature, China National Knowledge Infrastructure, National Digital Science Library, Korean Information Service System, and Korean Medical Database. Search terms were composed based on “Alzheimer” for participants and “Jihwangeumja” or “Dihuang Yinzi” for interventions and modified in Korean and Chinese (Tables 1–4).

**Table 1:** Search strategies for the PubMed.

No	Search items
#1	“Alzheimer Disease”[Mesh]
#2	alzheimer∗[Title/Abstract]
#3	AD[Title/Abstract]
#4	#1 OR #2 OR #3
#5	Jihwangeumja
#6	‘Dihuang Yinzi’
#7	#5 OR #6
#8	#4 AND #7

**Table 2:** Search strategy for the EMBASE.

No	Search items
#1	‘alzheimer disease’/exp
#2	alzheimer∗:ab,ti
#3	AD:ab,ti
#4	OR 1–3
#5	Jihwangeumja:ab,ti
#6	‘Dihuang Yinzi’:ab,ti
#7	OR 5–6
#8	#4 AND #7

**Table 3:** Search strategy for the CENTRAL.

No	Search items
#1	MeSH descriptor: [Alzheimer Disease] explode all trees
#2	alzheimer∗ or AD:ti,ab,kw (Word variations have been searched)
#3	#1 or #2
#4	Jihwangeumja:ab,ti
#5	‘Dihuang Yinzi’:ab,ti
#6	#4 or #5
#7	#3 and #6

**Table 4:** Search strategy for the CINAHL.

No	Search items
#1	MH “Alzheimer's Disease”
#2	TI alzheimer∗ OR AB alzheimer∗
#3	OR 1–2
#4	TI Jihwangeumja OR AB Jihwangeumja
#5	TI ‘Dihuang Yinzi’ OR AB ‘Dihuang Yinzi’
#6	OR 4–5
#7	#3 AND #6

CINAHL = Cumulative Index to Nursing and Allied Health Literature.

#### 2.3.2 Searching other resources

We will review the reference lists of the included studies to identify additional studies.

### 2.4 Data collection and analysis

#### 2.4.1 Study selection

**Figure 1:**
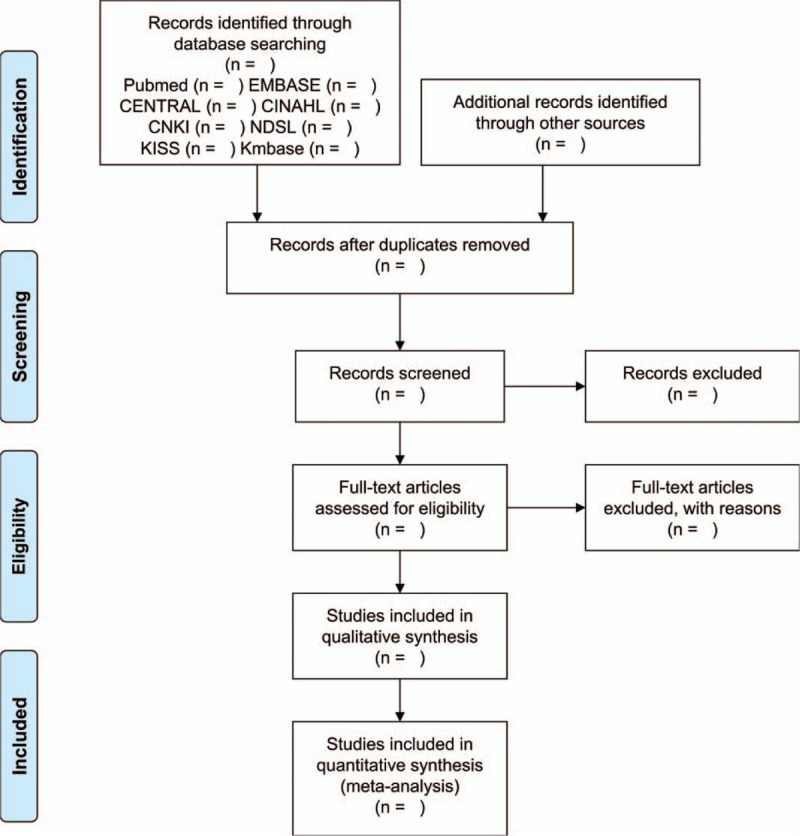
PRISMA flow diagram of literature screening and selection processes. PRISMA = preferred reporting items for systematic review and meta-analysis.

To select eligible studies for the systematic review and meta-analysis, studies identified through the database search will be assessed using the inclusion/exclusion criteria. Two researchers will independently screen studies with their titles and abstracts and then cross-check. The full text of studies that the 2 researchers cannot reach an agreement over will be reviewed by a third researcher to obtain their opinion. The selection will be performed according to the preferred reporting items for systematic reviews and meta-analysis flow chart shown in Figure 1.

#### 2.4.2 Data extraction and management

Two researchers will independently extract the study data and disagreements on the suitability of the data for inclusion will be resolved by reaching a consensus based on the opinion of the third researcher. We will use a standardized data extraction form that includes study design, intervention, comparison, duration, follow-ups, outcome measures, results (mean, standard deviations, and quartiles), and adverse events, and will be recorded using Microsoft Excel.

#### 2.4.3 Assessment of bias risk and quality of included studies

Two researchers will independently evaluate the risk of bias (ROB) in each included study using the Cochrane ROB tool. Moreover, the third researcher will mediate to resolve any conflicting opinions. This ROB tool includes random sequence generation, allocation concealment, blinding of participants, blinding of personnel, blinding of outcome assessment, selective reporting, and other biases. Each domain will be assessed as low, unclear, or high ROB. Conflicting opinions will be resolved with the help of the third researcher.

#### 2.4.4 Measurements of treatment effect

A meta-analysis will be conducted using RevMan version 5.4. Continuous data will be presented as the mean difference or standardized mean difference with a 95% confidence interval. For dichotomous data, the risk ratio with 95% confidence interval will be estimated and synthesized.

#### 2.4.5 Dealing with missing data

If there is any missing data, we will contact the authors to request the relevant information. If complete data are not available, we will report this in the assessment of bias risk, and only existing data will be used in the data analysis.

#### 2.4.6 Assessment of heterogeneity

The statistical heterogeneity will be evaluated using the *I*^2^ statistic, classified as follows: low, moderate, and high heterogeneity for *I*^2^ < 25%, 25% to 50%, and >75%, respectively.^[18]^

#### 2.4.7 Assessment of reporting biases

To assess the reporting bias visually, funnel plots were used if >10 trials were included in the meta-analysis.

#### 2.4.8 Data synthesis

Fixed-effects models will be used for the meta-analysis when there are ≤4 included studies. If there are >4 studies the random-effect model will be used, considering the heterogeneity.

## 3 Ethics and dissemination

No ethical approval is needed for this protocol because the data that will be used in this systematic review are not individual patient data. The results will be published in a peer-review journal and presented at a relevant conference.

## 4 Discussion

With the decline in the cognitive function and ability of AD patients to perform ADL, the physical, emotional, social, and financial burden of their caregivers increases.^[19]^ Unfortunately, current available drugs for treating AD do not have disease modifying effects and, therefore, novel treatment strategies are needed to treat AD. In 2020, a systematic review and meta-analysis of herbal medicine treatment for AD was conducted.^[20]^ The intervention we propose to investigate in this study includes a combination of various prescriptions and a single prescription from many countries. Various interventions are necessary for assessing the overall effectiveness of herbal medicine, but research on a single prescription is more necessary in actual clinical use.

Our research aims to increase the clinical applicability, including studies that used Jihwangeumja as the only intervention. Jihwangeumja has been used to treat high blood pressure, arteriosclerosis, stroke, and various age-related symptoms by reinforcing the function of the kidney.^[21]^ In Korean medicine, aging believed to be closely related to kidney deficiency and because Jihwangeumja reinforces the kidney, it is expected to exhibit a therapeutic effect in AD, where aging is the main etiological factor. In a previous study, Jihwangeumja improved the cognition and energy metabolism of mice by protecting the mitochondria from injury.^[22]^

Another study has shown that Jihwangeumja prevents antioxidant enzyme activities and regulates neuroinflammation by exerting neuroprotective effects on dopaminergic neurons and their microenvironment.^[23]^ Although the potential efficacy of Jihwangeumja in AD is expected to be mediated through these mechanisms, these results are limited to experimental studies and, therefore, clinical research studies would be necessary to confirm its effectiveness. We will conduct this systematic review of randomized controlled trials to verify the efficacy and safety of Jihwangeumja in AD patients. We expect to provide clinical evidence to support the use of Jihwangeumja as a novel possible treatment for AD and suggest further research through this review.

## Acknowledgment

The authors would like to thank Editage (www.editage.co.kr) for English language editing.

## Author contributions

**Conceptualization:** JaeYeong Lee, Ju Yeon Kim, In Chul Jung.

**Data curation:** Ji-Yoon Lee, Jin-Hyeong Jung.

**Formal analysis:** Jae Yeong Lee, Ju Yeon Kim.

**Funding acquisition:** In Chul Jung.

**Writing – original draft:** Jae Yeong Lee, Ju Yeon Kim.

**Writing – review & editing:** Jae Yeong Lee, Ju Yeon Kim, In Chul Jung.
